# Discrete mission planning algorithm for air-sea integrated search model

**DOI:** 10.1038/s41598-021-95477-7

**Published:** 2021-08-20

**Authors:** Yixiong Yu

**Affiliations:** School of Aeronautic Science and Engineering, Beihang University, Beijing, 100083 China

**Keywords:** Engineering, Mathematics and computing

## Abstract

The selection of optimal search effort for air-sea integrated search has become the most concerned issue for maritime search and rescue (MSAR) departments. Helicopters play an important role in maritime search because of their strong maneuverability and hovering ability. In this work, the requirements of maritime search were analyzed, from which a global optimization model with quantitative constraints for vessels and aircraft was developed by setting the least search time as single-objective optimization problem; then the improved Dinkelbach algorithm was used to solve the continuous programming problem, and the discrete mission planning algorithm was used to improve the calculation accuracy of search time and area. A case study shows that the errors in calculating search time and area decrease from 12–18 min to 36 s and from 76.5 to 0.45 n mile^2^, respectively. The results obtained from the discrete mission planning algorithm can provide better guidance for MASR departments in selecting optimal search scheme.

## Introduction

In the twenty-first century today, ports and channels are increasingly crowded with the rapid development of economic globalization and maritime shipping industry. Container ships develop towards large-scale and high-speed, and maritime traffic accidents occur frequently. The ability of maritime search and rescue (MSAR) is being severely tested^1^. Therefore, selecting the optimal search effort for MSAR becomes urgent issue to be solved for preventing serious loss of life and property associated with improper disposal of maritime traffic accidents.

At present, the new modes of air-sea integrated search and rescue with the participation of helicopters have received wide-spread attention. The advantages of applying helicopters to transport equipment, materials, rescue team members and rescued personnels are fast, efficient and less limited by geographical space. Many researchers have carried out studies on how to improve the mission effectiveness of helicopters and to scientifically select, plan and coordinate search effort.

Zhang et al. analyzed maritime emergency plans and established maritime emergency evaluation index system^2^. Five aspects (daily job, contingency plan, emergency rescue team, guarantee capability and technical support) were used to evaluate the system. Hao et al. evaluated maritime emergency management system to identify the contingency and defective factors^3^. A four-level indicator system was proposed and evaluated with the analytic hierarchy process and fuzzy comprehensive evaluation method. Jacobsen and Gudmestad suggested combining MSAR helicopters and multi-purpose emergency response vessels to improve the long-range rescue capability of Barents Sea operation^4^. They considered a way to provide search and rescue within 260 nautical miles and for at least 21 people in two hours. Xu et al. introduced an expert evaluation cloud model for MSAR capability^5^. Compared with the traditional fuzzy comprehensive evaluation method, the cloud model can provide more information. Zhang et al. presented a novel grey-cloud clustering comprehensive evaluation model for MSAR^6^. The improved grey-cloud whitening-weight function and improved analytic hierarchy process were used to establish the evaluation model, which can improve the reliability and accuracy of the evaluation results. Jia et al. used a four-layer weighted super-network and the indicator importance sort algorithm for constructing capability evaluation system of MASR^7^. Some aspects such as organization, equipment, project, technology, and their relationships to vessels were taken into account for the evaluation system. Recently, Ostermann et al. introduced a project to support the rescue forces at sea with unmanned aerial systems and thus to optimize the rescue process, which deals with the localization of potential accident sites, information of the rescuers and the provision of an efficient communication infrastructure^8^.

Above researches on MSAR evaluations mainly concentrated on maritime emergency response capability. Liu et al. recently evaluated the method for helicopter MSAR response plan with uncertainty^9^. An evaluation indicator system was extracted by analyzing uncertainty factors and mission flow. The Monte Carlo method was used for calculating the probability distribution and robustness of comprehensive emergency response plan, from which, the prototype system was built and evaluated. In addition, Liu et al. further used the particle swarm optimization algorithm and time–space weight for MSAR decision-making. A case simulation was carried out to test that the algorithm proposed can improve the success probability for the optimal MSAR mission area^10^.

Xing et al. established a global optimal model for search effort selection of MASR^11,12^. The continuous mission planning algorithm (CMPA) was used for solving the global optimization model. The deficiency of this algorithm is that the number of aircraft sorties is assumed to be a continuous variable. The over-simplified assumption may lead to a larger error in calculating time-consuming. In addition, the error range cannot be estimated when the approximate time interval for MASR is uncertain. In this work, to improve the calculation accuracy of search time and area, the author innovatively proposes a discrete mission planning algorithm (DMPA). This algorithm (1) assumes the aircraft and vessels spending the same time in the search task, (2) effectively makes up for the deficiency of the CMPA that assumes the number of aircraft sorties being a continuous variable, and (3) can improve the accuracy in calculating time-consuming for global optimal model of maritime search.

## Methods

### MASR mission model

**Figure 1:**
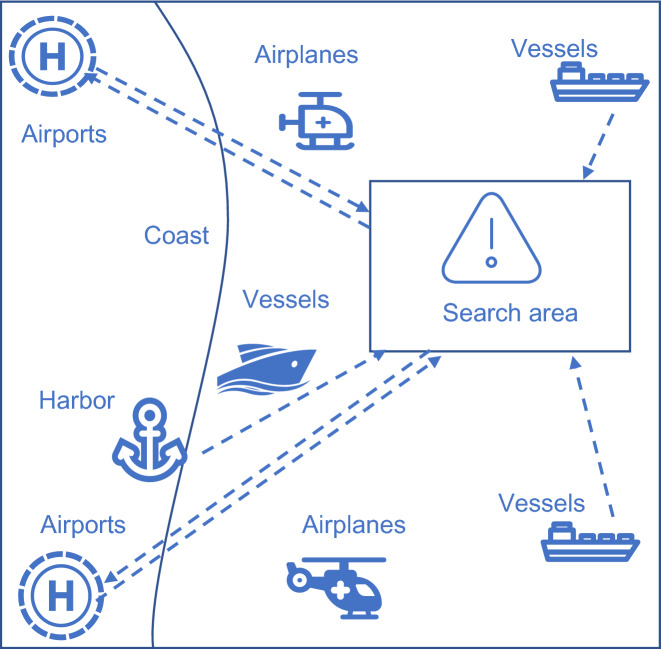
Schematic diagram of MASR task.

Figure 1 is the schematic diagram of MASR task, showing that there are multiple search and rescue professional helicopters and vessels around the sea area to be searched. In addition, some passing ships can be requisitioned. The basic parameters used in MASR mission model are introduced below.

In the maritime search, the search capability (*C*) of equipment is defined as:

1 $$ C = V \times W $$

Here *V* denotes search speed and *W* means scanning width^1^. The *V* values of helicopters and vessels are generally known and can be easily obtained. Their values in scanning width can be found in IAMSAR manual^1^, which are associated with the detection equipment, the type of targets to be searched, the search method and the environmental factors. When search equipment passes through a region containing many evenly distributed targets, *W* can be calculated with:

2 $$ W = \frac{M}{N \times V} $$

where *M* represents the number of targets found by maritime search equipment in unit time, and *N* means the number of targets per unit area. The values in scanning width of helicopters and vessels are given in reference^1^. To establish a global optimization model for maritime search, the following parameters are introduced:

- the sea area to be searched: *S* (n mile^2^);
- the total time of search operation: *T* (h);
- the number of aircraft near the sea area used for search: *P*;
- the number of vessels near the sea area used for search: *Q*;
- the maximum speed of the *i*th vessel: $V_{i}^{v}$ (Kn = n mile/h);
- the maximum speed of the *j*th aircraft: $V_{j}^{a}$ (Kn = n mile/h);
- the initial distance of the *i*th vessel: $D_{i}^{v}$ (n mile);
- the initial distance of the *j*th aircraft: $D_{j}^{a}$ (n mile);
- the maximum endurance time of the *j*th aircraft: $T_{j}^{d}$ (h).
- Then, the author can further deduce the secondary physical quantity:
- the time for the *i*th vessel rushing to the sea area: $\vec{T}_{i}^{v}$ (h);
- the time for the *j*th aircraft rushing to the sea area: $\vec{T}_{j}^{a}$ (h);
- the search time of the *i*th vessel in the sea area: $\overline{T}_{i}^{v}$ (h);
- the search time of the *j*th aircraft in the sea area: $\overline{T}_{j}^{a}$ (h);
- the search time of the *j*th aircraft in a complete flight: $\overline{T}_{j}^{ac}$ (h);
- the number of sorties of the *j*th aircraft: *F*_*j*_;
- the search capability of the *i*th vessel: $C_{i}^{v}$ (n mile^2^/h);
- the search capability of the *j*th aircraft: $C_{j}^{a}$ (n mile^2^/h).

In addition, to describe the call state of each vessel and each aircraft in search, *x*_*i*_ and *y*_*i*_ are introduced as 0–1 decision variables^11^:

3 $$ x_{i} = \left\{ {\begin{array}{ll} {{0}\;\;{\text{Ship}}\;{\text{i}}\;{\text{will}}\;{\text{not}}\;{\text{participate}}\;{\text{in}}} \\ {{1}\;\;{\text{Ship}}\;{\text{i}}\;{\text{will}}\;{\text{participate}}\;{\text{in}}\;\;\;\;} \\ \end{array} } \right. $$

4 $$ y_{i} = \left\{ {\begin{array}{ll} {{0}\;\;{\text{Airplane}}\;{\text{j}}\;{\text{will}}\;{\text{not}}\;{\text{participate}}\;{\text{in}}} \\ {{1}\;\;{\text{Airplane}}\;{\text{j}}\;{\text{will}}\;{\text{participate}}\;{\text{in}}\;\;\;\;} \\ \end{array} } \right. $$

When all the search efforts are taken into account, the sum of the search area becomes:

5 $$ S = \sum\limits_{i = 1}^{Q} {T_{i}^{v} C_{i}^{v} x_{i} } + \sum\limits_{j = 1}^{P} {T_{j}^{a} C_{j}^{a} y_{j} } $$

$C_{i}^{v}$ and $C_{j}^{a}$ are calculated with Eq. (1). Since there is no need to consider the vessel’s return time, $\overline{T}_{i}^{v}$ can be calculated with

6 $$ \overline{T}_{i}^{v} = T - \vec{T}_{i}^{v} $$

where *T* is the completion time of search operation and $\vec{T}_{i}^{v}$ is defined as

7 $$ \vec{T}_{i}^{v} = {{D_{i}^{v} } /{V_{i}^{v} }} $$

Xing et al. regarded the number of aircraft sorties (*F*_*j*_) as a continuous variable, which is calculated with^11,12^:

8 $$ F_{j} = {T / {T_{j}^{d} }} $$

Then $\overline{T}_{j}^{a}$ can be expressed with:

9 $$ \overline{T}_{j}^{a} = \overline{T}_{j}^{ac} \times F_{j} = {(}T_{j}^{d} - 2\vec{T}_{j}^{a} {)} \times {(}{T / {T_{j}^{d} {)}}} $$

The author defines the model established with this way as “continuous mission planning algorithm” (CMPA) model.

**Figure 2:**
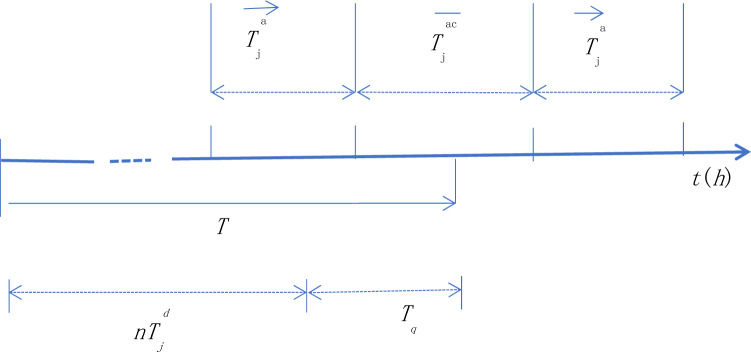
Time diagrammatic sketch used for aircraft search.

The author introduces a more accurate calculation method for $\overline{T}_{j}^{a}$. First, the remainder (*T*_q_) and quotient (*n*) of the mission completion time (*T*) divided by the endurance time of a single aircraft ($T_{j}^{d}$) are introduced, i.e. $T \div T_{j}^{d} = n \ldots T_{d}$. Their relationships are shown in Fig. 2.

Then the exact value $\overline{T}_{j}^{a}$ can be expressed as:

10 $$ \overline{T}_{j}^{a} = \left\{ {\begin{array}{ll} {n\overline{T}_{j}^{ac} \;\;\;\;\;\;\;\;\;\;\;\;\;\;\;\;\;\;\;\;\;\;\;\;0 < T_{q} < \vec{T}_{j}^{a} \;\;\;\;\;\;\;\;\;\;\;} \\ {n\overline{T}_{j}^{ac} + {(}T_{q} - \vec{T}_{j}^{a} {)}\;\;\;\;\;\;\;\vec{T}_{j}^{a} < T_{q} < {(}T_{j}^{d} - \vec{T}_{j}^{a} {)}} \\ {{(}n + {1)}\overline{T}_{j}^{ac} \;\;\;\;\;\;\;\;\;\;\;\;\;\;\;\;{(}T_{j}^{d} - \vec{T}_{j}^{a} {)} < T_{q} \;\;\;\;\;\;\;} \\ \end{array} } \right. $$

Similarly, the author defined the model based on Eq. (10) as “discrete mission planning algorithm” (DMPA) model. Obviously, the expressions of Eqs. (9) and (10) are different, which means the search times $\overline{T}_{j}^{a}$ calculated from CMPA and DMPA unequal. The simplification calculation based on CMPA will inevitably lead to a large error for the search time $\overline{T}_{j}^{a}$ of the *j*th aircraft, and the error range cannot be estimated when the approximate interval of completion time of maritime search is uncertain.

### Solvability of MASR models

For linear programming problems, the optimal solution may be a fraction or a decimal^13–16^. But for integer programming problems, the solutions must be an integer. The mission model of maritime search belongs to 0–1 integer programming. Since the variables *x*_*i*_ and *y*_*i*_ are limited to 0 or 1^17,18^. The author can obtain all possible combinations for *x*_*i*_ and *y*_*i*_ and find the optimal value. The number of possible schemes increases exponentially with increasing number of MASR equipment.

In the decision-making process of maritime search, the factors such as the emergency situation, the cost of search, and the contribution of search equipment may be taken into account in evaluating the optimal schemes. The models with constraints on the total number of aircraft and vessels become more significant for decision-makers. Therefore, quantitative constraints are added to search models with aircraft being *L*_*a*_ and vessels being *L*_v_. To realize the full coverage of the sea area to be searched, the following requirements should be met:

11 $$ \left\{ {\begin{array}{*{20}c} {S = \sum\limits_{i = 1}^{Q} {T_{i}^{v} C_{i}^{v} x_{i} } + \sum\limits_{j = 1}^{P} {T_{j}^{a} C_{j}^{a} y_{j} } } \\ {\sum\limits_{i = 1}^{Q} {x_{i} = L_{v} \;\;\;\;\;\;\;\;0 < L_{v} \le Q} } \\ {\sum\limits_{j = 1}^{P} {y_{i} = L_{a} \;\;\;\;\;\;\;\;0 < L_{a} \le P} } \\ \end{array} } \right. $$

Equation (11) can be converted to an optimization problem with time as a single-objective^12^:

12 $$ \left\{ {\begin{array}{*{20}c} {\min T = \frac{{\sum\limits_{i = 1}^{Q} {\vec{T}_{i}^{v} C_{i}^{v} x_{i} + S} }}{{\sum\limits_{i = 1}^{Q} {C_{i}^{v} x_{i} + \sum\limits_{j = 1}^{P} {{(}1 - {{2\vec{T}_{j}^{a} } / {T_{j}^{d} {)}C_{j}^{a} y_{j} }}} } }}} \\ {\sum\limits_{i = 1}^{Q} {x_{i} = L_{v} \;\;\;\;\;\;\;\;\;\;\;\;\;\;\;\;\;\;\;\;\;\;0 < L_{v} \le Q} } \\ {\sum\limits_{j = 1}^{P} {y_{i} = L_{a} \;\;\;\;\;\;\;\;\;\;\;\;\;\;\;\;\;\;\;\;\;\;0 < L_{a} \le P} } \\ \end{array} } \right. $$

In this work, the improved Dinkelbach algorithm^19–22^ was used to solve the Eq. (12). Equation (13) was introduced for linear fractional knapsack problem:

13 $$ \left. {\begin{array}{*{20}c} {{\text{max}}\;h{(}x{)} = \frac{{h_{1} {(}x{)}}}{{h_{2} {(}x{)}}} = \frac{{\sum\limits_{i = 1}^{n} {p_{i} x_{i} + p_{0} } }}{{\sum\limits_{i = 1}^{n} {q_{i} x_{i} + q_{0} } }}} \\ {\;\;\;\;\;\;\;\;\;\;\;\;\;\;\;\;\;\;\;\;\;\;\;\;\;\;\;\;\;\;\;\;x \in X} \\ \end{array} } \right\} $$

#### **Hypothesis**

*q*_*i*_ > 0; this hypothesis is necessary because of the requirement: $h_{2} {(}x{)} > 0$;

0 < *c*_*i*_ ≤ *d*; because *c*_*i*_ > *d* and $$x_{i}^{ * } = 0$$;

*p*_*i*_ > 0; if the coefficient of $x_{i}^{ * }$ is negative, then $x_{i}^{ * }$ must be zero;

$$ {{p_{1} } / {q_{1} }} \ge {{p_{2} } / {q_{2} \ge \cdots \ge {{p_{n} } / {q_{n} \ge {{p_{0} } / {q_{0} }}}}}} $$

According to the principle of Dinkelbach algorithm^19,20^, the optimization objective function can be constructed:

14 $$ G{(}\lambda {):}\;\;\left\{ {\begin{array}{*{20}c} {{\text{max}}\;g{(}x{)} = h_{1} {(}x{)} - \lambda h_{2} {(}x{)} = {(}p_{0} - \lambda q_{0} {)} + \sum\limits_{i = 1}^{n} {{(}p_{i} - \lambda q_{i} {)}x_{i} } } \\ {x \in X_{{0}} \;\;\;\;\;\;\;\;\;\;\;\;\;\;\;\;\;\;\;\;\;\;\;\;\;\;\;\;\;\;\;\;\;\;\;\;\;\;\;\;\;\;\;\;\;\;\;\;\;\;\;\;\;\;\;\;\;\;\;\;\;\;} \\ \end{array} } \right. $$

Some lemmas are used to assist in the construction of improved Dinkelbach algorithm^21,22^.

#### **Lemma 1**

*If* $p,\;q,\;r,s > 0$ *and* ${r /s} \ge {p /q}$ *, then* ${p/q} \le {{{(}\lambda p + \mu r{)}} / {\lambda q + \mu s) \le {r / s}}}$ *when* $\lambda ,\;\mu \ge 0$ *and* $\lambda + \mu > 0.$ *If and only if* $\mu = 0\;{(}\lambda = 0)$ *(or* ${r /s} = {p/q}$ *), the equality will hold.*

#### **Lemma 2**

*If,*$p,\;q,\;r,\;s,\;t,\;u > 0$, ${p / q} < {r / s} < {t /u}$* and*${{{(}p + t{)}} / {{(}q + u{)}}} \le {{{(}p + r{)}} / {{(}q + s{)}}}$*, then*$t \le r$.

#### **Lemma 3**

*If*$p,q,r,s > 0$, ${p / q} \le {r / s}$,* andq* < *s*, then ${r / s} \le {{{(}r - p{)}} / {{(}s - q{)}}}$.* If and only if* ${p / q} = {r / s}$,* the equality holds.*

The conclusions of Lemmas 1 to 3 can be used to deduce Theorem V.

#### **Theorem V**

*If a feasible solution*$\overline{x} = {(}\overline{x}_{1} ,\overline{x}_{2} , \ldots ,\overline{x}_{n} {)}$*satisfies*$h{(}\overline{x}{)} \ge {{p_{l} } / {q_{l} }}$, *then there must be an optimal solution,*$x^{*} = {(}x_{1}^{*} ,x_{2}^{*} , \ldots ,x_{n}^{*} {)}$, *satisfying*$x_{i}^{*} = 0,\;i = l,\;l + {!},\; \ldots ,\;n$.

**Figure 3:**
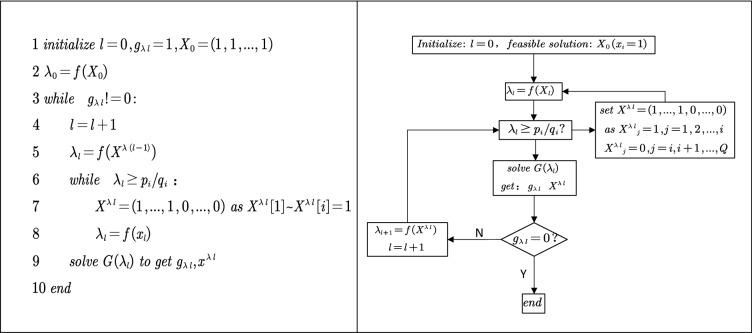
Flowchart and pseudocode of improved Dinkelbach algorithm.

Figure 3 shows the flowchart and pseudocode of improved Dinkelbach algorithm with Theorem V introduced:

Step 1: Set any feasible solution $x_{0} {(}x_{i} = 1,\;i = 1,\;2,\; \ldots ,\;Q{)}$ and *l* = 0; turn to Step 2.
Step 2: Set $\lambda_{i} = f{(}x_{l} {)}$; turn to Step 3.
Step 3: Iterate through ${{p_{i} } / {q_{i}^{{}} }}$, and judge whether *λ*_*l*_ is greater than ${{p_{i} } / {q_{i}^{{}} }}$. If so, turn to Step 6; if not, turn to Step 4.
Step 4: Solve *G*(*λ*_*l*_) and obtain the optimal solution *G*(*λ*_*l*_) and the optimal value *g*_*λl*_. If, *g*_*λl*_ = 0 then *x*^*λl*^ is the optimal solution of the original problem (F), and *λ*_*l*_ is its final value. Solving terminates. Otherwise, turn to Step 5.
Step 5: Let $\lambda_{l + 1} = f{(}x^{\lambda l} {)}$ and $l = l + 1$, turn to Step 3.
Step 6: Let each of the first *i* terms of *x*^*λl*^ be equal to 1 and each of last (*Q*-*i*) terms equals to 0. Return to Step 2.

To intuitively apply Dinkelbach algorithm for search mission model and its improved solution, the equivalent transformation of the model is firstly carried out with hypothesis:

15 $$ \left\{ {\begin{array}{ll} {p_{0} = \sum\limits_{j = 1}^{P} {{(}1 - 2{{\vec{T}_{j}^{a} } / {T_{j}^{d} {)}C_{j}^{a} y_{j} }}} } \\ {p_{i} = C_{i}^{v} ,\;\;i = 1,\; \ldots ,\;Q\;\;\;\;\;\;\;\;} \\ {q_{0} = S\;\;\;\;\;\;\;\;\;\;\;\;\;\;\;\;\;\;\;\;\;\;\;\;\;} \\ {q_{i} = \vec{T}_{i}^{v} C_{i}^{v} ,\;i = 1,\; \ldots ,\;Q\;\;\;\;} \\ \end{array} } \right. $$

Then the model can be expressed as follows:

16 $$ \left\{ {\begin{array}{ll} {{\text{max}}\;T = \frac{{\sum\limits_{i = 1}^{Q} {p_{i} x_{i} + p_{0} } }}{{\sum\limits_{i = 1}^{Q} {q_{i} x_{i} + q_{0} } }}\;\;\;\;} \\ {{{\text{s}}}.{{\text{t}}}.\;\sum\limits_{i = 1}^{Q} {x_{i} = L_{v} \;\;\;1 \le L_{v} \le Q} } \\ \end{array} } \right. $$

The following conclusions can be obtained from the known conditions and the characteristics of the model parameters:

17 $$ S > 0,\;\vec{T}_{i}^{v} C_{i}^{v} > 0,\;C_{i}^{v} > 0\;\;{(}i = 1,\; \ldots ,\;Q{)} $$

The author can derive:

18 $$ q_{0} > 0,\;q_{i} > 0,\;p_{i} > 0\;\;\;\;{(}i = 1,\; \ldots ,\;Q{)}\; $$

Under the total constraint of $\sum\limits_{j = 1}^{P} {y_{j} } = L_{a}$, $0 < L_{a} \le Q$, the decision variable *y*_*i*_ is set as 1. In the implementation of the algorithm, the author lets $A_{j} = {(}1 - 2{{\vec{T}_{j}^{a} } / {T_{j}^{d} {)}C_{j}^{a} }}$ and the decision variable *y*_*j*_ being 0 with the constraint of *A*_*j*_ < 0. Sort *A*_*j*_ from large to small, and then renumber them by the subscripts *j*. By the total amount constraint *L*_*a*_, the decision variable *y*_*j*_ of first *L*_*a*_ terms is set as 1:

19 $$ \left\{ {\begin{array}{*{20}c} {T_{j}^{d} < 2\vec{T}_{j}^{a} \;\;\;\;y_{j} \equiv 0\;\;\;\;\;\;\;\;\;\;} \\ {T_{j}^{d} > 2\vec{T}_{j}^{a} \;\;\;\;y_{i} = \left\{ {\begin{array}{*{20}c} {1\;j \le L_{a} } \\ {0\;j > L_{a} } \\ \end{array} } \right.} \\ \end{array} } \right. $$

$\sum\limits_{i = 1}^{Q} {p_{i} } x_{i} + p_{0}$ regarded as *f*_1_ and $\sum\limits_{i = 1}^{Q} {p_{i} } x_{i} + q_{0}$ as *f*_2_, can be substituted into Dinkelbach algorithm to obtain the value of decision variables *x*_*i*_ through iterative solution method. By substituting *p*_*i*_, *q*_*i*,_ *p*_0_, and *q*_0_ into the improved Dinkelbach algorithm, the value of decision variable *x*_*i*_ can also be solved.

**Figure 4:**
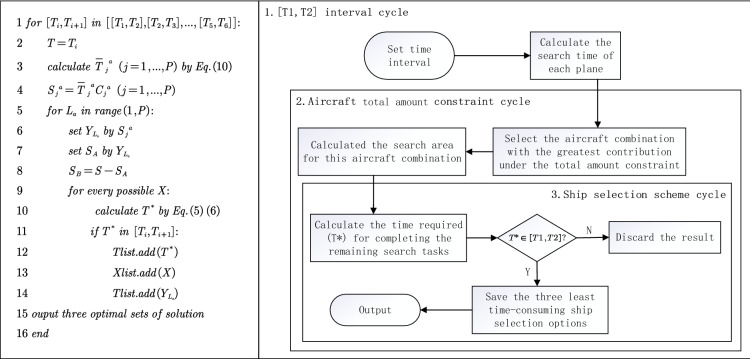
Flowchart and pseudocode of discrete mission planning algorithm.

To improve the calculation accuracy of search time and area, the DMPA proposed in this work is used to find the optimal solution by traversing the three physical quantities: possible time interval, aircraft quantity constraint, and vessel combination. Figure 4 shows the flowchart and pseudocode of DMPA:

Step 1: Give a time interval [*T*_1_, *T*_2_] and take *T*_1_ as the mission completion time for the aircraft. $\overline{T}_{j}^{a}$ (h) can be obtained according to Eqs. (6)–(10) and the search area can be calculated with:
  20 $$ S_{i}^{a} = \overline{T}_{j}^{a} C_{j}^{a} $$
Step 2: Give aircraft quantitative constraint; rank the search area *S*_*i*_^a^ in descending order; select the aircraft combination scheme that can contribute the largest search area; and determine the value of decision variables *y*_*i*_.
Step 3: Traverse all the vessel dispatching schemes and determine *x*_*i*_ value. The vessel quantitative constraint is not considered. The time T^*^ required for the vessel searching *S*_*B*_ under each possible scheme can be calculated. When T^*^ falls into the interval of [*T*_1_, *T*_2_], it is considered as the qualified solution. All the qualified T^*^ are sorted and the smallest three groups of results are selected for output.
Step 4: Enter the next cycle [*T*_1_, *T*_2_] and repeat Steps 1 to 3. Each operation includes searching the qualified T^*^ in five continuous and equal time intervals.

## Results and discussion

### Simulation conditions

Hypothesis: A fishing boat carrying 10 people is missing and waiting for search. The meteorological visibility of the sea area is 1.3 n mile and the wind power is 4 kts. The probability of fishing vessels being found in the sea area to be searched is equal. According to the activity log of the fishing area, the following information is known: the location of center point being (26.77N, 120.66E) and area being (2000 n mile^2^). The available search facilities, together with their search parameters are shown in Tables S1 and S2 in the Supplementary information.

### Calculation results from CMPA and DMPA

Table S3 in Supplementary information shows the calculation results from CMPA^11,12^, from which a preliminary search based on DMPA is carried out near the minimum value of 4.05 h. Table S4 in Supplementary information shows the search results when the initial time point *T*_1_ = 4.0 and the step *h* = 0.1 are set. After that, a more accurate search is carried out near the values of 4.30–4.39 h. Table 1 shows the search results with the initial time of *T*_1_ = 4.37 and the step of *h* = 0.01.

**Table 1:** Precise results from DMPA with *T*_1_ = 4.37 and *h* = 0.01.

No	Time interval		Aircraft		Vessels	Time consuming
	T1	T2	Aggregate constraint	Options	Options	
S_a_	4.37	4.38	3	1,2,3	1,2,4,6,7,8,9,10	4.37255
S_b_	4.38	4.39	3	1,2,3	1,3,4,5,6,7,9,10	4.38536
S_c_	4.39	4.40	3	1,2,3	2,3,4,5,6,8,9,10	4.39445
S_d_			3	1,2,3	1,3,4,5,7,8,9,10	4.39462
S_e_	4.40	4.41	3	1,2,3	2,4,5,6,7,8,9,10	4.40289
/	4.41	4.42	No possible scheme			

### Scheme analysis

The number of schemes obtained from the CMPA is always determined by the total number of aircraft and vessels. In this case, the total number of schemes is 10 (vessels) × 3 (aircraft) = 30 (see Table S3 in Supplementary information). However, the number of schemes from DMPA is related to time interval, and decreases with the approximation of time interval to minimum time and reduction of time interval range. When the time interval approaches the minimum time and the interval length is set as 0.01 h, there are only five feasible schemes (see Table 1).

It should be noted that when Dinkelbach algorithm is used, the condition of jumping out of the loop is not set to *g*_λ_ = 0, but to the minimum numerical interval − 10^–9^ to 10^–9^ near 0. It is proved that there is no need to further narrow the convergence interval. With the reduction of time interval and the increase of iteration times, the actual combination schemes will not change.

For the DMPA, the error of search time for aircraft is controlled within 0.01 × 60 = 0.6 min = 36 s, and the error of search area is within 4.11 n mile^2^, when the time interval is reduced to 0.01 h. For this time interval, the decision-makers can take the results from DMPA as the exact solution.

The same selection schemes from CMPA and DMPA are extracted to compare their time-consume. When the initial time point is set as *T*_1_ = 4.0 and the step *h* = 0.2, three schemes from each algorithm can be found in Tables 2 and 3.

**Table 2:** Three schemes from CMPA.

Scheme	Aircraft options	Vessel options	Time consuming (h)
D29	1,2,3	1,2,3,4,5,6,8,9,10	4.14
D27	1,2,3	1,2,3,4,5,6,10	4.35
D24	1,2,3	1,2,4,5	4.76

**Table 3:** Three schemes from DMPA.

Scheme	Aircraft options	Vessel options	Time consuming (h)
E1	1,2,3	1,2,3,4,5,6,8,9,10	4.3784
E2	1,2,3	1,2,3,4,5,6,10	4.5856
E3	1,2,3	1,2,4,5	4.9032

As can be seen in Table 2, the three schemes, D29, D27 and D24 all are beyond the interval given by the DMPA, i.e. the time ranges of [4.20, 4.38], [4.40, 4.59] and [4.80, 4.90] respectively corresponding to E1, E2 and E3 in Table 3. Table S5 in Supplementary information shows the searching area of equipment in D30 scheme on CMPA^11,12^. It can be seen that the total search area is 1923.49 n mile^2^. Because of the actual value being 2000 n mile^2^, the absolute error is 76.5 n mile^2^ and the relative error is 3.8%. When the search sea area increases, the error increases. However, the total contribution of search area of S_a_ scheme on DMPA is 1999.557053 n mile^2^, and the error is only 0.45 n mile^2^. Obviously, the DMPA based on time intervals can find the relatively optimal scheme, which produce smaller calculation errors in time-consuming and search area. As the no free lunch theorem suggests, an algorithm that can be well suited to an optimization problem may not always work effectively on other problems^23,24^. The DMPA cannot find the optimal result only via several operations on reducing time interval.

## Conclusions

To overcome the deficiency in the CMPA that regarded the number of aircraft sorties as a continuous variable, the author in this paper innovatively proposed the DMPA for solving the mission model of maritime search with quantitative constraints for vessel and aircraft. The DMPA assumes that the number of aircraft sorties as discrete, which can produce smaller calculation errors in time-consuming (36 s) and search area (0.45 nmile^2^). The DMPA proposed in this work provides a new theoretical basis for the field of MSAR planning algorithm.

## Supplementary Information

Supplementary Information.
