# Supplementary material for: Discrete mission planning algorithm for air-sea integrated search model
Source: Sci Rep. 2021 Aug 20;11:16957. doi: 10.1038/s41598-021-95477-7 (PMC8379196; doi:10.1038/s41598-021-95477-7)
Supplement: Supplementary file 1 — Supplementary Information. [file 41598_2021_95477_MOESM1_ESM.pdf]

Supplementary information

Discrete mission planning algorithm for air-sea integrated search model

Yixiong Yu*

School of Aeronautic Science and Engineering, Beihang University, Beijing 100083, China

*Corresponding author: Yixiong YU, email: yuyixiong@buaa.edu.cn; Tel.: +86 10 65367590;

Fax: 86 10 65367590; ORCID: 0000-0002-9999-5938

Tables

No.	Initial distance (n mile)	Maximum velocity (n mile/h)	Velocity correction	Wind correction	Maximum duration (h)	Sweep width (km)	Search capability (n mile^2/h)
1	27.73	126.89	1	1	3.8	1.023	129.84
2	34.33	118.79	1	1	3.2	1.023	121.55
3	48.03	156.05	0.9	1	3.5	1.023	159.68

Table S1. Information on search effort from aircraft

No.	Initial distance (n mile)	Maximum speed (n mile/h)	Sweep width (km)	Search capability (n mile^2/h)
1	8.96	20.1	1.023	20.57
2	15.89	20.1	1.023	20.57
3	19.87	18	1.023	18.42
4	35.21	30	1.023	30.70
5	26.14	20.1	1.023	20.57

6	27.59	20.1	1.023	20.57
7	25.32	18	1.023	18.42
8	26.49	18.58	1.023	19.01
9	31.35	20.1	1.023	20.57
10	38.76	22	1.023	22.51

Table S2. Information on search effort from vessels

**No.**	**Quantitative constraint**		**Time consuming**	**Aircraft serial number**	**Vessel serial number**
	Aircraft	Vessels			
D1	1	1	12.55	3	4
D2	1	2	11.18	3	1,4
D3	1	3	10.13	3	1,2,4
D4	1	4	9.30	3	1,2,4,10
D5	1	5	8.63	3	1,2,4,5,10
D6	1	6	8.07	3	1,2,4,5,6,10
D7	1	7	7.61	3	1,2,4,5,6,9,10
D8	1	8	7.22	3	1,2,3,4,5,6,9,10
D9	1	9	6.88	3	1,2,3,4,5,6,8,9,10
D10	1	10	6.58	3	1,2,3,4,5,6,7,8,9,10
D11	2	1	7.35	1,3	4
D12	2	2	6.87	1,3	1,4

D13	2	3	6.48	1,3	1,2,4
D14	2	4	6.16	1,3	1,2,4,5
D15	2	5	5.89	1,3	1,2,4,5,10
D16	2	6	5.64	1,3	1,2,4,5,6,10
D17	2	7	5.44	1,3	1,2,3,4,5,6,10
D18	2	8	5.25	1,3	1,2,3,4,5,6,9,10
D19	2	9	5.08	1,3	1,2,3,4,5,6,8,9,10
D20	2	10	4.93	1,3	1,2,3,4,5,6,7,8,9,10
D21	3	1	5.40	1,2,3	4
D22	3	2	5.15	1,2,3	1,4
D23	3	3	4.93	1,2,3	1,2,4
D24	3	4	4.76	1,2,3	1,2,4,5
D25	3	5	4.61	1,2,3	1,2,4,5,6
D26	3	6	4.48	1,2,3	1,2,3,4,5,6
D27	3	7	4.35	1,2,3	1,2,3,4,5,6,10
D28	3	8	4.24	1,2,3	1,2,3,4,5,6,9,10
D29	3	9	4.14	1,2,3	1,2,3,4,5,6,8,9,10
D30	3	10	4.05	1,2,3	1,2,3,4,5,6,7,8,9,10

Table S3. Calculation results from the CMPA

No.	Time	Aircraft	Vessels	Time

	**interval**					**consuming**
/	T1	T2	Quantitative constraint	Options	Options	
/	4.0	4.1	No possible scheme			
/	4.1	4.2	No possible scheme			
/	4.2	4.3	No possible scheme			
L1	4.3	4.4	3	1,2,3	2,3,4,5,6,7,8,9,10	4.30
L2					1,2,3,5,6,7,8,9,10	4.39
L3	4.4	4.5	3	1,2,3	3,4,5,6,7,8,9,10	4.48
L4					1,2,3,5,6,8,9,10	4.48
L5					2,3,4,5,7,8,9,10	4.41

Table S4. Preliminary results of DMPA with T_1 = 4.0 and h = 0.1

**No.**	**Velocity**	**Initial distance**	**Search capability**	**Search time**	**Search area**
A1	126.89	27.73	129.84	3.39	440.71
A2	118.79	34.33	121.55	3.18	386.90

A3	156.05	48.03	159.68	3.13	499.24
V1	20.10	8.96	20.57	3.60	74.13
V2	20.10	15.89	20.57	3.26	67.04
V3	18.00	19.87	18.42	2.95	54.26
V4	30.00	35.21	30.70	2.88	88.30
V5	20.10	26.14	20.57	2.75	56.55
V6	20.10	27.59	20.57	2.68	55.06
V7	18.00	25.32	18.42	2.64	48.69
V8	18.58	26.49	19.01	2.62	49.89
V9	20.1	31.351	20.56733	2.49	51.22
V10	22	38.75953	22.5115	2.29	51.51
Total search area					1923.49

Table S5. Searching area of equipment in D30 scheme from CMPA
